# Exogenous α-Tocopherol Regulates the Growth and Metabolism of Eggplant (*Solanum melongena* L.) under Drought Stress

**DOI:** 10.3390/plants12020237

**Published:** 2023-01-04

**Authors:** Nudrat Aisha Akram, Rohina Bashir, Gulshan Ashraf, Shehnaz Bashir, Muhammad Ashraf, Mohammed Nasser Alyemeni, Andrzej Bajguz, Parvaiz Ahmad

**Affiliations:** 1 Department of Botany, Government College University, Faisalabad 38040, Pakistan; 2 Institute of Molecular Biology and Biotechnology, University of Lahore, Lahore 54590, Pakistan; 3 Botany and Microbiology Department, King Saud University, Riyadh 11451, Saudi Arabia; 4 Department of Biology and Plant Ecology, Faculty of Biology, University of Bialystok, Ciolkowskiego 1J, 15-245 Bialystok, Poland; 5 Department of Botany, Government Degree College, Pulwama 192301, Jammu and Kashmir, India

**Keywords:** antioxidants, drought stress, eggplant, mitigation, α-tocopherol

## Abstract

The present investigation was designed to improve drought stress tolerance in eggplant (*Solanum melongena* L.) through the exogenous application of α-tocopherol (TOC). For exogenous application, two modes, i.e., foliar spray (FS) and pre-sowing seed treatment (PS), were used. Water deficiency treatment (50% field capacity (FC)) was applied on 32-day-old seedlings of two eggplant cultivars, i.e., Janak and Black Beauty. Five levels of TOC (0 mg/L, 50 mg/L PS, 100 mg/L PS, 50 mg/L FS, and 100 mg/L FS) were applied as PS and FS. Pre-sowing seed treatment was conducted before seed sowing, while FS treatment after 30 days of drought stress treatment. After 15 days of TOC as an FS application, it was observed that drought stress significantly reduced plant growth (5–15%) and chlorophyll contents (4–10%), while it increased proline (4–6%), glycine betaine (GB) (5–10%), malondialdehyde (MDA) (10.8%), hydrogen peroxide (15–16%), relative membrane permeability (RMP) (5–8%), and the activities of peroxidase (7–8%) and superoxide dismutase (12–15%) in both eggplant cultivars. The TOC application (FS and PS) exhibited a positive role in overcoming the adverse effect of water stress on eggplants. Plant growth increased (15–18%) as a result of the application of TOC, which could be linked with improved chlorophyll, ascorbic acid (AsA), GB, proline, total soluble proteins (TSP), and the activities of peroxidase (POD) and superoxide dismutase (SOD) activities. The reactive oxygen species H_2_O_2_ was also decreased by TOC application. Overall, TOC as a foliar spray was more effective in improving the accumulation of proline, GB, AsA, and activities of SOD and POD enzymes, while PS treatment was more effective in reducing RMP and improving the TSP of eggplant. Cv. Black Beauty was comparatively better in root dry weight, chlorophyll *a* and *b*, and MDA contents, while cv. Janak in RMP, AsA, TSP, and activity of the POD enzyme. It can be inferred that the application of TOC was useful in counteracting the harmful effects of drought stress on both cultivars of eggplants.

## 1. Introduction

The global production and human population need to be balanced to meet the demand of 2050 [1]. Although in the last few decades, crop productivity has improved significantly, this increase is inadequate for the population requirement. According to an estimate, crop productivity is reduced on average by about 50% under natural conditions due to various stresses [2]. Of the different constraints prevalent across the world, water scarcity is believed to be the major factor in the failure of crop sustainability, especially in dryland areas [3,4]. Plants can naturally adopt a variety of mechanisms to cope with extreme environments [5]. These include alterations at the molecular, biochemical, and cellular levels, including the synthesis of osmoprotectants, secondary metabolites, plant growth regulators, antioxidant production, soluble sugars, and activation/deactivation of protein cascades [6,7,8]. These modulations induce the regulation of stress tolerance and various defense responses [9].

Exogenously applied ascorbic acid (AsA), tocopherols, proline, GB, dopamine, catechins, etc., induce a significant role in minimizing the effects of abiotic stresses [10,11,12]. These substances can be used exogenously as seed, growth, or foliage treatments. Seed soaking in water or other vital chemicals is a promising method to promote seedling and germination growth under control and stress clues [13,14]. Previous studies reveal that seed soaking with 0.05 mM and 10 mM α-tocopherol (TOC) increased the level of antioxidants and proline in *Leymus chinensis* [15]. Similar results have been reported in salt-stressed sunflower plants subjected to TOC [16]. In addition to seed priming, the application of certain chemicals to plant leaves is valuable for improving crop growth and productivity [17]. These chemicals can easily enter the leaf through the cuticle, stomata, and entire leaf surface [18]. It suggests that foliar applied 500 mg/L TOC considerably improved the vegetative and physio-biochemical parameters of soybean under salinity stress [19]. Similarly, Sadiq et al. [20] reported the effectivity of TOC on the mung bean under drought conditions. The seed priming, as well as foliar spray of alpha-tocopherol, has been reported to be an effective technique to protect plants under abiotic stresses [21]. TOC is a lipophilic antioxidant having low molecular weight. Its accumulation is tissue-specific in plants [22]. In plants, tocopherol synthesis occurs in the chloroplast, and enzymes involved in this process are present in the inner membrane of the chloroplast [23]. TOC has been documented to function synergistically with antioxidants such as glutathione, carotenoids, and ascorbate under stress conditions [24]. These low-molecular-weight conjugated antioxidants play a positive role against stress damage [25]. Thus, externally applied TOC is an easy and efficient method to enable plants to tolerate stress conditions [20,26].

The eggplant (*Solanum melongena* L.), which represents the Solanaceae family [27], is widely grown in several countries, commonly in China, India, Sri Lanka, Bangladesh, and Pakistan [28]. Of these, China is the major grower, producing 50.19 million tons of brinjal [29]. It is a huge source of income for poor farmers and a cheap summer vegetable for ordinary people. It is rich in amino acids, minerals, vitamins, anthocyanins, and phenolic compounds [30,31]. Due to the higher level of phenolic compounds, it can counteract oxygen radicals under stress conditions [32]. As water is vital for brinjal growth at every stage of its life cycle, lack of water can affect plant growth and production [33]. Therefore, the current experiment has been planned to study the effectivity of exogenously applied TOC on eggplant growth and key physiological and biochemical parameters such as the oxidative defense system and osmoregulation under water stress conditions. For this, the following hypotheses were tested: (1) TOC has a stimulating effect on the growth and selected metabolites in eggplant; (2) TOC overcomes the repressive impact of water stress.

## 2. Results

**Figure 1:**
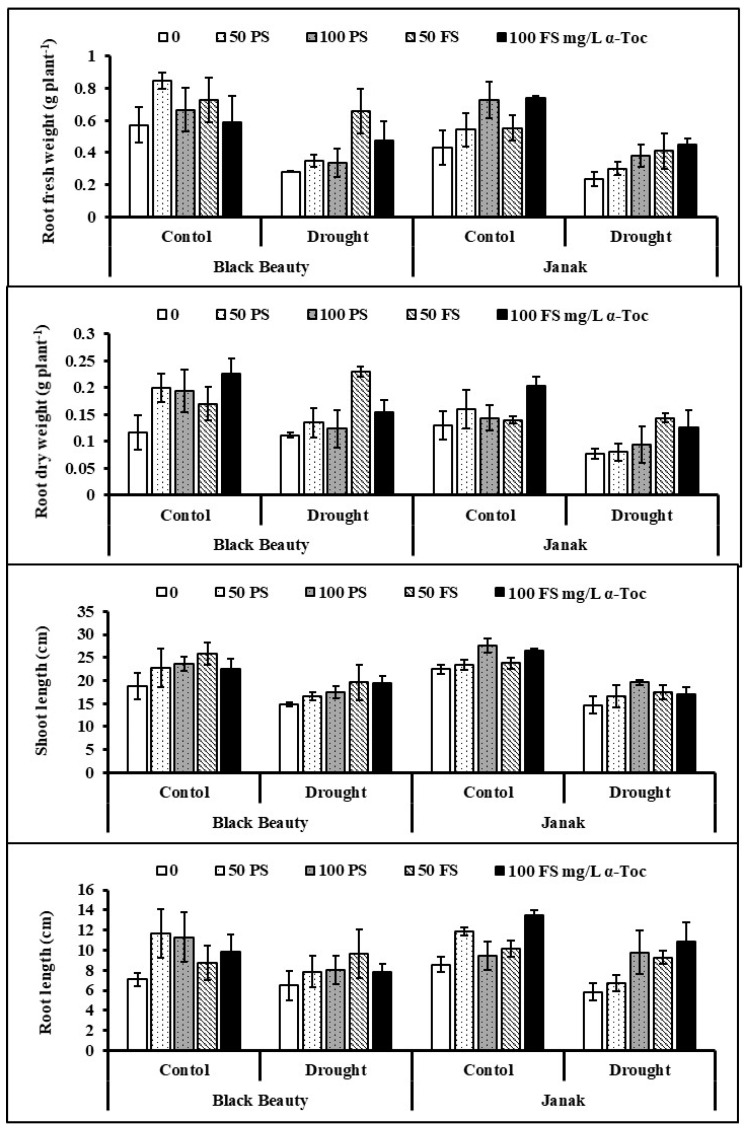
Fresh root weights, dry root weights, and lengths of eggplants subjected to exogenous TOC (pre-sowing (PS) and foliar spray (FS)) under varying water regimes (mean ± S.E.; *n* = 4).

Data showed that imposition of water stress (50% FC) markedly suppressed the fresh and dry weights of the roots of both eggplant cultivars, Black Beauty and Janak. Externally applied TOC in the form of seed priming and foliar spray indicated a considerable increasing impact on fresh and dry biomasses of both cultivars of eggplant under control and water-limited stress. Overall, 50 mg/L TOC, both as a seed soaking and foliar spray applications, was more effective than 100 mg/L, particularly for cv. Black Beauty under stress regime (Table 1; Figure 1). However, both cultivars were the same in fresh root weight, but cv. Black Beauty performed better than cv. Janak in terms of dry root weight under water stress regimes.

Water stress considerably reduced the lengths (root and shoot) of both eggplant cultivars. Exogenously applied TOC through seed priming and foliar spray significantly (*p* < 0.01) improved these attributes of eggplant. Plants treated with TOC improved the lengths of shoots and roots (Table 1; Figure 1). The responses of both eggplant cultivars were almost the same to water stress and TOC treatments.

**Figure 2:**
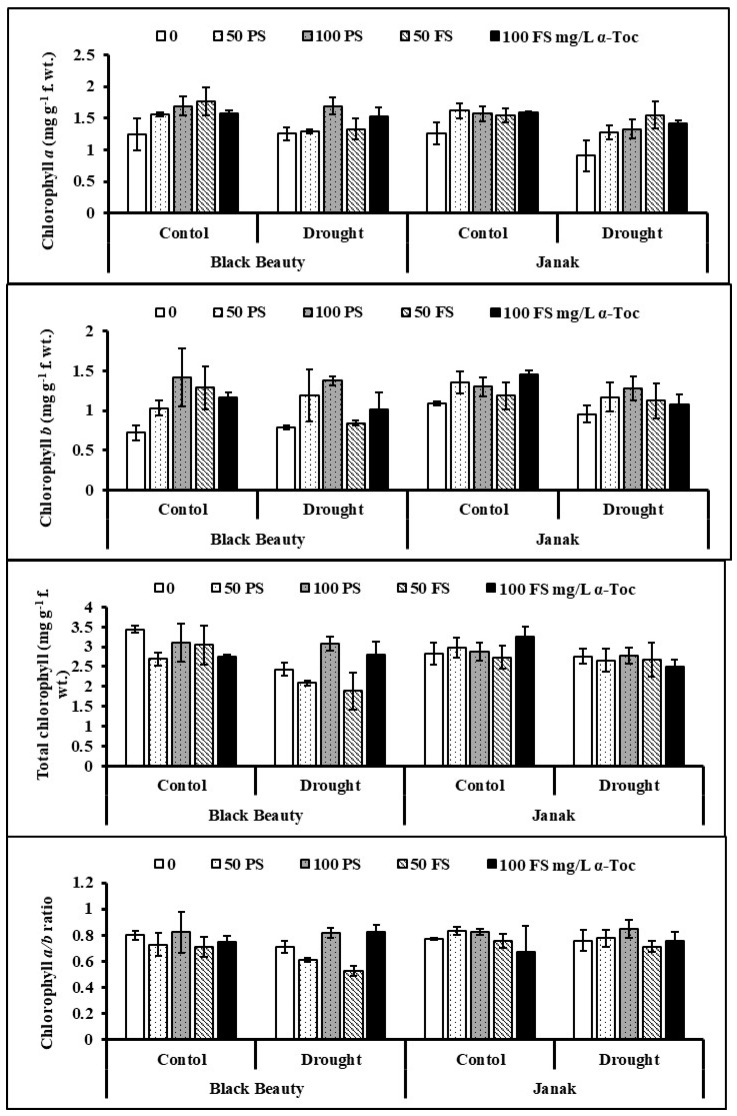
Leaf chlorophyll content in two eggplant cultivars subjected to exogenous (pre-sowing (PS) and foliar spray (FS)) under varying water regimes (mean ± S.E.; *n* = 4).

Water stress induced a prominent decline in the chlorophyll pigments of both eggplant cultivars. The effect of TOC application was significantly increasing on chlorophyll *a* and *b* contents. Seed priming (100 mg/L) was most effective in increasing these pigments under water deficit stress, particularly in the case of cv. Black Beauty (Figure 2). It was observed that both eggplant cultivars were the same in response to exogenously applied TOC and water regimes.

**Figure 3:**
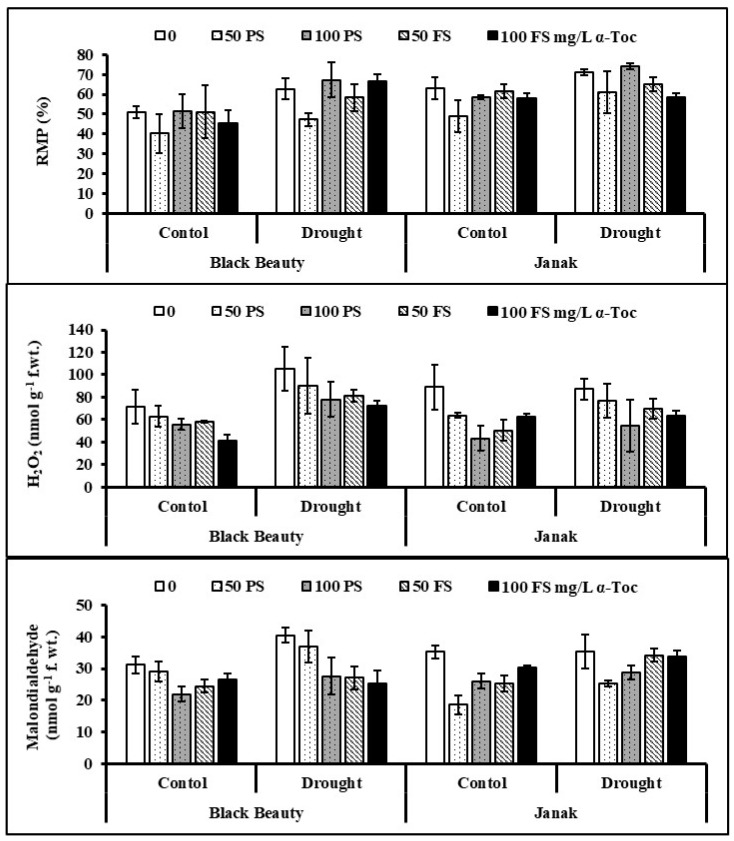
Leaf relative membrane permeability (RMP), hydrogen peroxide (H_2_O_2_), malondialdehyde (MDA) content in two cultivars of eggplants subjected to exogenous TOC (pre-sowing (PS) and foliar spray (FS)) under varying water regimes (mean ± S.E.; *n* = 4).

The relative membrane permeability (RMP) of both eggplant cultivars increased considerably (*p* < 0.01) due to drought conditions. Treatment of the seeds with TOC, particularly 50 mg/L, was better in decreasing the RMP of stressed eggplants (Figure 3). The cv. Janak was higher in RMP than in cv. Black Beauty under varying water regimes.

Hydrogen peroxide (H_2_O_2_) increased markedly (*p* < 0.01) under water-limited stress in both eggplant cultivars. TOC was found to be effective in reducing the overproduction in water-stressed plants of both eggplant cultivars (Table 1; Figure 3). Treatment with 100 mg/L TOC as seed priming and through the foliage was the most effective in minimizing the H_2_O_2_ contents of stressed eggplants. However, the response of both eggplant cultivars was the same in this attribute.

The level of malondialdehyde (MDA) was enhanced noticeably (*p* < 0.01) in water-stressed eggplants (Figure 3). However, TOC as seed soaking, as well as the foliar spray, was effective in decreasing the MDA contents in both eggplant cultivars. Overall, 100 mg/L TOC was found to be better in reducing the MDA content under water stress. The difference was found between both cultivars and cv. Black Beauty was relatively higher in MDA content under drought stress.

**Figure 4:**
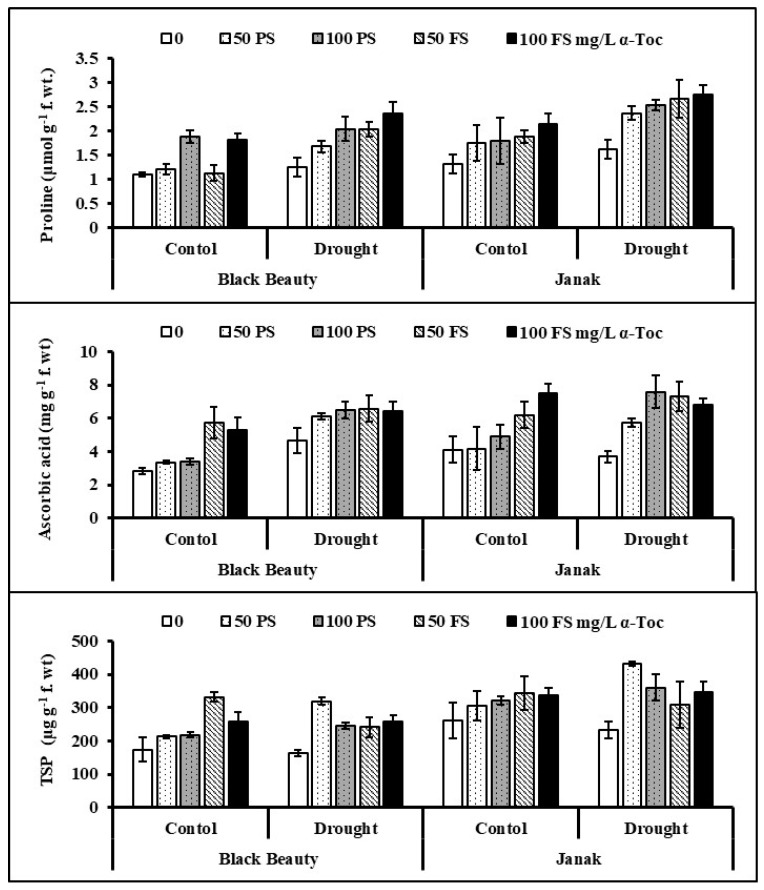
Leaf glycine betaine, proline, ascorbic acid, and total soluble protein (TSP) contents in two cultivars of eggplants subjected to exogenous TOC (pre-sowing (PS) and foliar spray (FS)) under varying water regimes (mean ± S.E.; *n* = 4).

High concentrations of potential osmoprotectants, such as proline and glycine betaine, were found in both eggplant cultivars under water stress (Figure 4). TOC application, particularly as a foliar spray (100 mg/L), was better in improving these attributes of eggplants. Both eggplant cultivars responded differentially; however, the higher accumulation of GB and proline under stress conditions was observed in cv. Janak.

Water stress considerably improved the AsA contents in both eggplant cultivars (Figure 4). TOC treatments had a positive impact on ascorbic acid concentration in water-stressed eggplants. Of all tocopherol treatments, foliage spray (50 and 100 mg/L) increased AsA accumulation in both eggplant cultivars under water stress. The cv. Black Beauty was lower in AsA accumulation under varying water regimes.

Total soluble proteins (TSP) remained unaffected in both cultivars under water stress. However, TOC treatment was effective in improving TSP in cv. Janak is under water stress. Of all TOC levels, seed priming with 50 mg/L was promising in enhancing TSP concentrations in both cultivars under water deficit stress. Of both eggplant cultivars, cv. Black Beauty is lower in TSP contents under water stress conditions (Figure 4).

**Figure 5:**
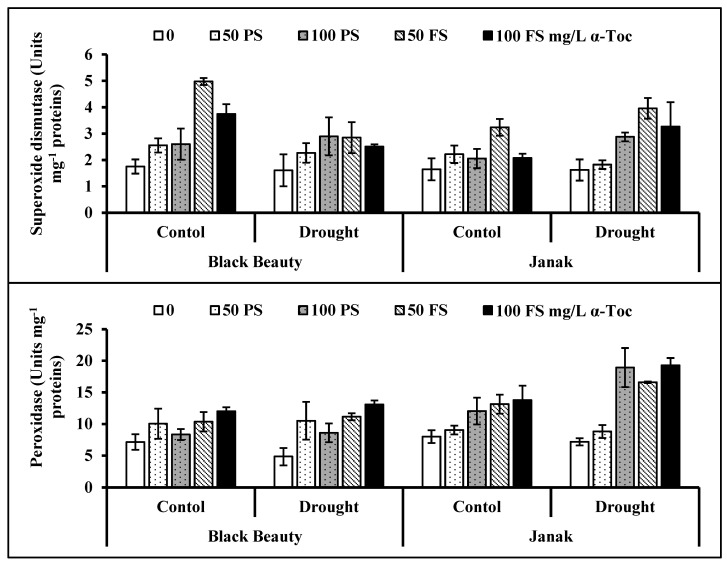
Activities of superoxide dismutase and peroxidase enzymes in two cultivars of eggplant leaves subjected to exogenous TOC (pre-sowing (PS) and foliar spray (FS)) under different water regimes (mean ± S.E.; *n* = 4).

Data revealed that the activity of superoxide dismutase (SOD) was not affected, while the activity of the peroxidase (POD) enzyme was improved in the eggplant cultivars under water stress (Table 1; Figure 5). A foliar spray of TOC was effective in increasing SOD and POD activities, and 100 mg/L TOC as an FS was more influential in increasing the activities of these enzymes in eggplants. The activity of SOD was the same in both eggplant cultivars, while cv. Janak was better in POD activity under water stress.

**Figure 6:**
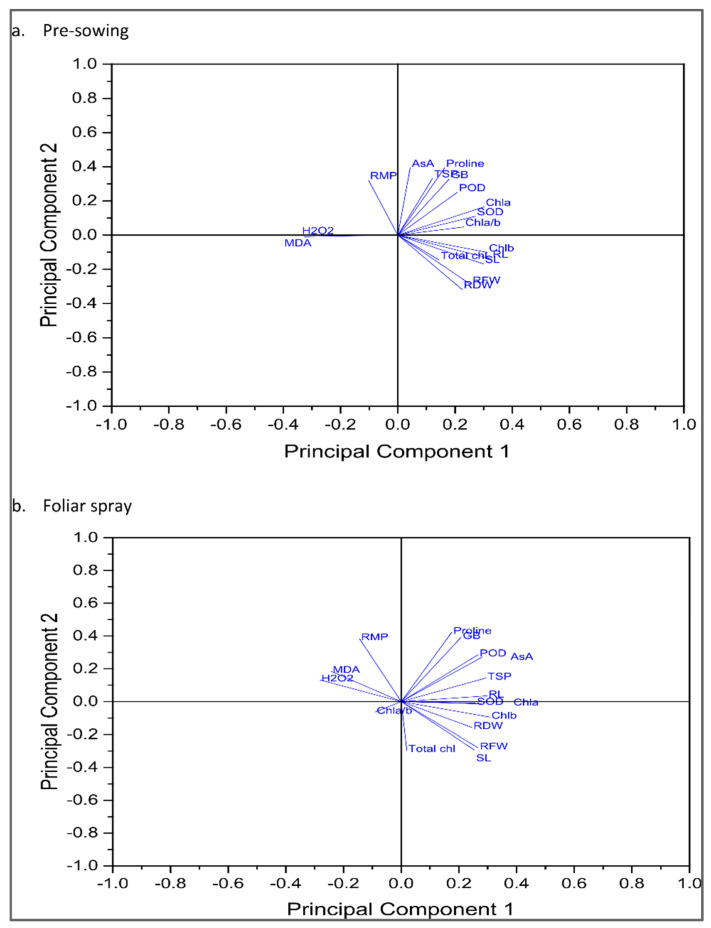
Principal component analysis of eggplants subjected to exogenous ((**a**) pre-sowing (PS) and (**b**) foliar spray (FS)) application of TOC under water deficit (50% FC) and control (100% FC) conditions (*n* = 4).

**Figure 7:**
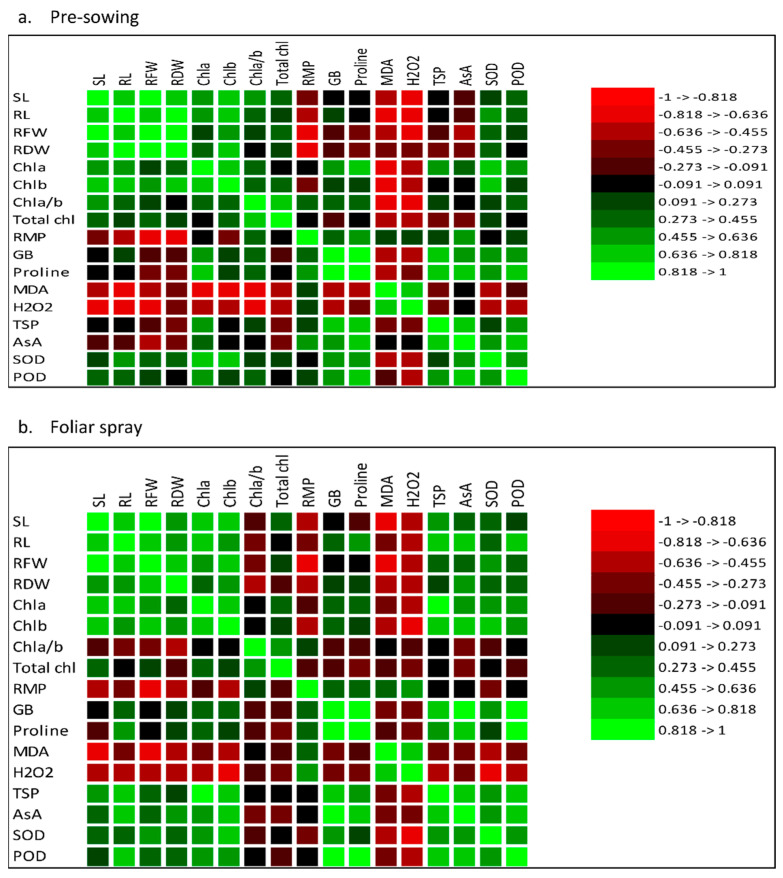
Correlation analysis among different attributes of eggplants subjected to exogenous ((**a**) pre-sowing (PS) and (**b**) foliar spray (FS)) application of TOC under water deficit conditions (50% FC) and control (100% FC) (*n* = 4).

The correlation and analysis of the principal components showed that plant morphological characteristics of the plants, including the growth of plants, were positively associated with an improved oxidative defense system under water stress and TOC foliage spray (Figure 6 and Figure 7).

## 3. Discussion

Water scarcity is the main abiotic stress that influences the growth and yield of crop plants. To minimize the adversaries of water stress, agricultural scientists use the exogenous application of various chemicals such as phytohormones and antioxidants. TOC is also one of the antioxidants used to improve tolerance to stress in crops. It is an antioxidant that helps to stabilize membrane stability and functions during growth and metabolism by eliminating reactive oxygen species under stress conditions [21,34]. Chemicals can be applied as a pre-sowing seed treatment, applied to foliage, and added to the growth medium [35]. In this work, foliar spray and pre-sowing seed treatment with TOC of eggplant under water stress showed the positive effects of exogenously applied TOC. There is no other report on the role of TOC on eggplant growth and metabolism under water deficit stress. In this study, TOC application significantly increased plant biomass, but both cultivars were uniform in this attribute. These results are similar to those of Sadiq et al. [36] in mung bean and *Vicia faba* [34], and *Hibiscus rosasineses* [37]. These authors attributed that TOC application enhanced the antioxidant capacity of plants.

An increase in plant biomass is directly correlated with photosynthetic efficiency and assimilation in plants, which, in turn, are connected with photosynthetic pigments and plant water relations, which are considered vital factors to effectively regulate plant development. We found that TOC application enhanced the levels of photosynthetic pigments in both eggplant cultivars, but cv. Black Beauty showed a better response compared to cv. Janak. Both modes of TOC application, i.e., foliar and seed priming, showed a similar effect on photosynthetic pigments. TOC-induced improvement in chlorophyll pigments was also observed in salt-stressed wheat plants [38]. An improvement in growth and yield production under stress conditions is associated with an improved plant antioxidative defense system that helps stabilize cellular membranes from the damage of ROS. Among ROS, H_2_O_2_ is one of the powerful signaling molecules and an oxidant within cellular membranes, hindering various metabolic actions [39]. We observed that water stress improved H_2_O_2_ content, but TOC influenced the reduction of H_2_O_2_ overproduction, and both eggplant cultivars examined in the present study exhibited a similar response. Furthermore, H_2_O_2_ overproduction can enhance MDA concentration under water stress [35]. Water scarcity in our study improved MDA levels in both eggplant cultivars. Oxidative stress-induced lipid peroxidation is common in plants under abiotic stresses [40]. In the current investigation, TOC application significantly reduced H_2_O_2_ and MDA levels in eggplants. Tocopherol applications suppressed the effect of oxidative stress by removing peroxyl radicals responsible for lipid peroxidation [34].

For ROS detoxification, enzymatic (POD, SOD, ascorbate peroxidase, and catalase) and non-enzymatic (tocopherols, AsA, and phenolics) antioxidants are significantly triggered [35,41,42]. We examined that SOD activity remained unaffected under stress conditions, whereas POD was higher under water-deficit stress. The TOC enhanced the activities of the SOD and POD enzymes. The SOD activity was similar in both cultivars, while the POD activity was greater in cv. Janak than the other cultivar under water stress regimes. These findings can be related to those of Ye et al. [43], who reported that Carex leucochlora treatment with 0.8 mM tocopherol improved plant growth by scavenging ROS and strengthening the antioxidative defense mechanism under non-stress and stress regimes.

Ascorbic acid (AsA) is considered to be beneficial even in small concentrations, as it can effectively suppress the adverse influence of salinity stress by scavenging free radicals [44]. In our study, the application of tocopherol caused an improvement in ascorbic acid in both eggplant cultivars. Similar results were found in onions due to tocopherol under stress regimes [45]. An increase in GB and proline content occurs in stressed plants [46]. These osmolytes are known to protect cellular structures from the adverse effects of stress [47]. In our study, tocopherol priming and foliar applications showed a significant effect on increasing the concentrations of GB and proline under stress conditions. The cultivar Janak showed a comparatively better response under stress conditions. These findings are in accordance with Al Hassan et al. [48], who found increased GB levels in tomato plants.

## 4. Materials and Methods

### 4.1. Experimental Layout

A trial was carried out in the summer season of 2019 in Faisalabad, Pakistan. The eggplant seeds (cv. Black Beauty and Janak) were provided by the Research Institute located in Faisalabad, Pakistan. The seeds were sterilized with sodium hypochlorite (5%) before sowing in plastic pots, each containing 8.0 kg of soil. In all pots, 5 plants/pot were arranged with four replicates. During the experimentation, the average temperature, 27.2–38.3 °C (night and day temperature); sunshine, 9.0 h; humidity, 69%; soil, sandy loam soil with pH 7.8 were determined. The plants were subjected to well-watered and well-watered (control) conditions. The control pots were irrigated several times a week to maintain soil moisture near field capacity (FC), while the stress pots experienced soil drying by withholding irrigation until they reached 50% FC. The water deficit, that is, 50% FC along with the control (100% FC), was used on the 32-day-old seedlings. Both eggplant cultivars were subjected to exogenous application of α-tocopherol (TOC) through presoaking of seeds as well as a foliar spray. After 30 days of drought stress treatment, TOC levels (50 and 100 mg/L) were applied as a foliar application to 62-day-old plants. For seed priming, seeds were seeded at the above-mentioned concentrations of TOC mentioned above for 15 h before sowing in the soil. The remaining procedure of the water stress treatment was the same as in the case of the foliar application of TOC. In addition, TOC, along with 0.01% Tween-20, was used for foliar application with a plastic manual sprayer. After two weeks of TOC foliage treatment, the readings were determined for the morphological, biochemical, and physiological parameters of 77-day-old plants. All chemicals used in the present study were obtained from certified companies, including BDH, AnalaR, England, Merck, Germany, MP Biomedicals, LLC, Illkirch, France.

### 4.2. Morphological Parameters

For the measurement of the lengths and the weights of both the shoots and roots, two plants were harvested from each treatment. The samples were dried by setting an oven (Memmert, GmbH, Schutzart, IN30) at 72 °C. Fresh and dry weights were recorded using an analytical balance (Shimadzu, Kioto, Japan).

### 4.3. Chlorophyll Determination

The procedure described by Wellburn [49] was used for the estimation of chlorophyll content by reading all sample extracts at 480, 645, and 663 nm on a spectrophotometer (Model Hitachi-U 2001, Tokyo, Japan).

### 4.4. Relative Membrane Permeability

Following Yang et al. [50], a leaf (each 0.5 g) was chopped and dipped in 10 mL of distilled water. All samples were vortexed for half an hour, and electrical conductivity (EC) using an EC meter (TDS-P10M, Bioevopeak, Co., Ltd., Shandong, China) as EC0. The samples were kept overnight and then recorded as EC1. After that, these samples were autoclaved for 2 h, and EC2 was recorded. Relative membrane permeability (RMP) was calculated on a percentage basis.

### 4.5. Hydrogen Peroxide Determination

Leaves (0.5 g) were harvested, immediately frozen in liquid nitrogen, ground, and powder stored at −80 °C until the H_2_O_2_ determination assay [51]. Frozen powder (150 mg) was directly homogenized with 1 mL of a solution containing 0.25 mL 0.1% trichloroacetic acid (TCA), 0.5 mL 1M potassium iodide and 0.25 mL 10 mM potassium phosphate buffer (pH 7) at 4 °C for 10 min. At the same time, for each sample, a control was prepared with H_2_O instead of KI for the tissue coloration background. The homogenate was centrifuged at 12,000× *g* for 15 min at 4 °C. Then, 200 μL of the supernatant from each tube was placed in UV-microplate wells and left to incubate at room temperature (20–22 °C) for 20 min. The optical density (OD) was measured at 390 nm.

### 4.6. Malondialdehyde Determination

The MDA content was recorded adopting the Cakmak and Horst [52] using fresh leaves (0.5 g) and extracted in 1% TCA. Then, thiobarbituric acid (3 mL; 0.5%) was added, and OD was noted at 532 nm and 600 nm.

### 4.7. Determination of Glycine Betaine

For the determination of GB [53], the dry leaf material (0.5 g) was occasionally shaken in 10 mL toluene (0.5%). One mL of the filtrate was reacted with 1 mL of 2N sulfuric acid. Then this extract (0.5 mL) was taken in a test tube, and KI3 solution was added. The contents were cooled in a chiller. Then, 2.8 mL of ice-cooled de-ionized H_2_O and 5 mL of 1,2-di-chloroethane were added to the reaction mixture, and OD was observed at 365 nm.

### 4.8. Free Proline Determination

Leaves (0.5 g) were homogenized using sulfosalicylic acid (10 mL; 3%) and filtered following the method of Bates et al. [54] method. Filtrate (2 mL), acid ninhydrin (2 mL), and glacial acetic acid (2 mL) were mixed. The mixture was heated in a water bath (W2OL2, Sheldon Manufacturing Inc., Cornelius, OR, USA) at 60 °C for 1.0 h and cooled before adding toluene (4 mL). After mixing, two layers were prominent, and the absorbance of the upper colored layer was observed at 520 nm.

### 4.9. Ascorbic Acid Determination

Leaves (0.5 g) were chopped in TCA (6%; 10 mL) [55]. Then, 1.0 mL of filtrate was homogenized with thiourea (1.0 mL; 10%), dinitrophenyl hydrazine (2 mL; 2%), and the mixture was boiled. After cooling, 5 mL of H_2_SO_4_ was added, and OD was noted at 530 nm.

### 4.10. Determination of Total Soluble Proteins

Following the Bradford [56] method, leaves (250 mg) were extracted in a K buffer (pH 7) and centrifuged at 12,000× *g* for 15 min. The filtrate and Bradford reagent were mixed, and absorbance was noted at 595 nm.

### 4.11. Determination of Enzyme Antioxidants

Ice-chilled leaves (500 mg) were extracted in 50 mM phosphate buffer (pH 7.8). Then the mixture was centrifuged at 4 °C for 10 min, and the aliquot was preserved at −20 °C to determine enzyme activity. The POD activity was determined by preparing the reaction mixture following the Chance and Maehly [57] method. The SOD activity was recorded by adopting the Beauchamp and Fridovich [58], Giannopolitis, and Ries [59] methods. Each reaction mixture was composed of leaf extract, riboflavin, nitroblue tetrazolium chloride, EDTA, methionine, and phosphate buffer (pH 7.8), followed by recorded OD of the mixture at 560 nm.

### 4.12. Statistical Analysis

A completely randomized experiment using three factors, that is, drought stress, cultivars, and exogenously applied TOC, along with four replicates. Data were analyzed (ANOVA) using the CoStat software. The comparison between mean values was determined at a significance level using the LSD test. Furthermore, the principal component analysis and correlations among different parameters were determined.

## 5. Conclusions

In summary, drought stress significantly suppressed plant growth and chlorophyll content while it increased proline, glycine betaine, malondialdehyde, H_2_O_2_, relative membrane permeability, and activities of peroxidase and superoxide dismutase enzymes in both eggplant cultivars. However, exogenously applied TOC as a foliar spray or seed treatment improved eggplant growth and physio-biochemical characteristics under water stress. However, foliar application of TOC is more effective than pre-sowing in the accumulation of proline, GB, AsA, and activities of SOD and POD enzymes. Of both eggplant cultivars, cv. Black Beauty performed better in root dry weight, chlorophyll *a* and *b,* MDA contents, and cv. Janak in RMP, AsA, TSP, and activity of POD enzyme. The improvement in plant growth of eggplants subjected to water-deficit stress was related to improved chlorophyll pigments and accumulation of osmoprotectants and antioxidants. However, more research is suggested in the future on different cultivars of eggplant or other crops to assess the effects of exogenous application of TOC on plant yield production.

## Figures and Tables

**Table 1:** Mean square values (ANOVA) for the growth and physiological, biochemical characteristics of eggplant (*Solanum melongena* L.) subjected to exogenous application of alpha-tocopherol (TOC) under drought stress.

Source of Variations	df	Shoot Length	Root Length	Root Fresh Weight
**Cultivars (Cv)**	1	8.588 ns	8.214 ns	0.080 ns
**Drought stress (D)**	1	609.9 ***	59.20 **	0.954 ***
**TOC**	4	38.56 *	20.95 *	0.078 *
**Cv × D**	1	24.19 ns	0.770 ns	0.001 ns
**Cv × TOC**	4	10.70 ns	6.761 ns	0.048 ns
**D × TOC**	4	0.633 ns	8.092 ns	0.033 ns
**Cv × D × TOC**	4	5.360 ns	4.151 ns	0.020 ns
**Error**	48	11.72	6.812	0.027
		**Root dry weight**	**Chlorophyll *a***	**Chlorophyll *b***
**Cultivars (Cv)**	1	0.019 **	0.202 ns	0.113 ns
**Drought stress (D)**	1	0.025 ***	0.215 **	0.512 **
**TOC**	4	0.009 **	0.330 *	0.331 **
**Cv × D**	1	0.001 ns	0.021 ns	0.022 ns
**Cv × TOC**	4	0.001 ns	0.057 ns	0.037 ns
**D × TOC**	4	0.005 *	0.049 ns	0.018 ns
**Cv × D × TOC**	4	7.555 ns	0.061 ns	0.071 ns
**Error**	48	0.001	0.087	0.063
		**Chlorophyll *a/b* ratio**	**Total chlorophyll**	**RMP**
**Cultivars (Cv)**	1	0.027 ns	0.077 ns	905.5 **
**Drought stress (D)**	1	0.017 ns	2.473 *	1568 ***
**TOC**	4	0.035 ns	0.326 ns	343.7 *
**Cv × D**	1	0.015 ns	0.324 ns	82.76 ns
**Cv × TOC**	4	0.021 ns	0.229 ns	37.32 ns
**D × TOC**	4	0.018 ns	0.135 ns	42.23 ns
**Cv × D × TOC**	4	0.002 ns	0.459 ns	69.67 ns
**Error**	48	0.017	0.243	122.4
		**GB**	**Proline**	**H_2_O_2_**
**Cultivars (Cv)**	1	467.9 **	2.840 ***	476.7 ns
**Drought stress (D)**	1	445.1 **	4.235 ***	4851 **
**Alpha tocopherol (TOC)**	4	336.6 ***	1.531 ***	1829 *
**Cv × D**	1	41.41 ns	0.096 ns	1386 ns
**Cv × TOC**	4	1.680 ns	0.132 ns	256.2 ns
**D × TOC**	4	42.79 ns	0.149 ns	18.12 ns
**Cv × D × TOC**	4	27.72 ns	0.049 ns	137.3 ns
**Error**	48	50.33	0.149 ns	488.1
		**MDA**	**TSP**	**AsA**
**Cultivars (Cv)**	1	0.701 ns	1026 ***	7.575 *
**Drought stress (D)**	1	316.3 **	3122ns	28.55 ***
**TOC**	4	166.1 ***	2290 ***	15.52 ***
**Cv × D**	1	0.877 ns	891.4 ns	4.423 ns
**Cv × TOC**	4	139.4 **	2280ns	0.920 ns
**D × TOC**	4	15.78 ns	1323 **	3.691 *
**Cv × D × TOC**	4	29.09 ns	559.6 ns	0.794 ns
**Error**	48	28.80	3006	1.368
		**SOD**	**POD**	
**Cultivars (Cv)**	1	1.332 ns	141.8 ***	
**Drought stress (D)**	1	0.212 ns	33.92 *	
**TOC**	4	7.363 ***	109.1 ***	
**Cv × D**	1	5.081 **	31.53 *	
**Cv × TOC**	4	0.073 ns	29.39 **	
**D × TOC**	4	0.645 ns	14.32 ns	
**Cv × D × TOC**	4	1.416 ns	5.875 ns	
**Error**	48	0.567	7.509	

*, **, and *** = significant at the levels of 0.05, 0.01, and 0.001, respectively; ns = not significant.

## Data Availability

Not applicable.

## Abbreviations

AsA	ascorbic acid
FC	field capacity
FS	foliar spray
GB	glycine betaine
H_2_O_2_	hydrogen peroxide
MDA	malondialdehyde
POD	peroxidase
PS	pre-sowing
RMP	relative membrane permeability
ROS	reactive oxygen species
SOD	superoxide dismutase
TOC	α-tocopherol
TSP	total soluble protein
